# Metabolomics reveals the protective of Dihydromyricetin on glucose homeostasis by enhancing insulin sensitivity

**DOI:** 10.1038/srep36184

**Published:** 2016-10-31

**Authors:** Liang Le, Baoping Jiang, Wenting Wan, Wei Zhai, Lijia Xu, Keping Hu, Peigen Xiao

**Affiliations:** 1 Institute of Medicinal Plant Development, Chinese Academy of Medical Sciences, Peking Union Medical College, No. 151 Malianwa North Road, Haidian District, Beijing 100193, P. R. China; 2 State Key Laboratory of Bioactive Substances and Resources Utilization of Chinese Herbal Medicine, Ministry of Education, No. 151 Malianwa North Road, Haidian District, Beijing 100193, P. R. China.; 3 Institute of Chinese Materia Medica, China Academy of Chinese Medical Sciences, Beijing 100700, P. R. China.

## Abstract

Dihydromyricetin (DMY), an important flavanone found in Ampelopsis grossedentata, possesses antioxidative properties that ameliorate skeletal muscle insulin sensitivity and exert a hepatoprotective effect. However, little is known about the effects of DMY in the context of high-fat diet (HFD)-induced hepatic insulin resistance. Male Sprague-Dawley(SD) rats were fed a HFD(60% fat) supplemented with DMY for 8 weeks. The administration of DMY to the rats with HFD-induced insulin resistance reduces hyperglycemia, plasma levels of insulin, and steatosis in the liver. Furthermore, DMY treatment modulated 24 metabolic pathways, including glucose metabolism, the TCA cycle. DMY significantly enhanced glucose uptake and improved the translocation of glucose transporter 1. The specificity of DMY promoted the phosphorylation of AMP-activated protein kinase (AMPK). In addition, the exposure of HepG2 cells to high glucose concentrations impaired the insulin-stimulated phosphorylation of Akt2 Ser474 and insulin receptor substrate-1 (IRS-1) Ser612, increased GSK-3β phosphorylation, and upregulated G6Pase and PEPCK expression. Collectively, DMY improved glucose-related metabolism while reducing lipid levels in the HFD-fed rats. These data suggest that DMY might be a useful drug for use in type 2 diabetes insulin resistance therapy and for the treatment of hepatic steatosis.

Insulin resistance can lead to hyperinsulinemia, an impairment of insulin-regulated glucose homeostasis that is a key pathogenic factor in obesity, metabolic syndrome, and type 2 diabetes mellitus (T2DM)^1^. Hepatic insulin resistance is a hallmark of type 2 diabetes^2^. Dietary excesses appear to be a particularly important contributor to the high prevalence of insulin resistance among Western and Westernized populations. Unfortunately, defects in glucose metabolism and the underlying mechanisms controlling diet-induced insulin resistance are only partially understood.

In many studies of diet-related insulin resistance, a rodent model is generated by feeding with an artificial diet containing excessive amounts of fat, commonly supplying 60% of calories derived from fat rather than the usual 10% in standard rodent chow. The chronic consumption of a high-fat diet (HFD) can result in an abnormal accumulation of fat in the liver and can trigger toxic effects that contribute to the pathogenesis of type 2 diabetes and the related metabolic syndrome^3^,^4^. HFD-fed rats and mice typically exhibit impairments in insulin-stimulated hepatic Akt activity^5^,^6^, which leads to a decrease in hepatic glycogen synthesis due to decreased activation of glycogen synthase-3β (GSK3β)^7^. Impairments in hepatic Akt activity also result in decreased phosphorylation of the forkhead box OX-1 protein (FoxO1), which mediates the effect of insulin/Akt on gluconeogenic enzymes [i.e., increases in expression of phosphoenolpyruvate caroxykinase (PEPCK) and glucose-6-phosphatase (G6Pase)]^8^,^9^, resulting in exaggerated glucose release via GLUT2. Thus, increases in hepatic glucose output contribute to insulin resistance in rats and mice.

Previous reports have demonstrated the translocation of GLUT2 to the plasma membrane via the AMP-activated protein kinase (AMPK) pathway in the liver^10^. AMPK acts as an energy sensor to control glucose and lipid metabolism^11^,^12^. The activation of AMPK results in increased lipid and glucose catabolism and fatty acid metabolism, whereas reduced glucose production^13^ has a beneficial effect on glucose homeostasis and peripheral insulin sensitivity^14^,^15^.

*Ampelopsis grossedentata* (Hand. -Mazz) W. T. Wang grows wild in the southern region of China. A tea made from its stems and leaves has been used for several hundred years by the Yao people in the Guangxi and Hunan provinces of China to treat common colds and pyretic fever, a painful swelling of the pharynx and larynx, as well as jaundice hepatitis^16^,^17^. Dihydromyricetin (DMY; also called Ampelopsin) is one of the most prominent flavonoids isolated from the stems and leaves of *Ampelopsis grossedentata*, comprising over 27% of the stems and leaves of vine tea^18^ and over 40% of the cataphyll^19^. DMY exerts numerous pharmacological activities, including cough relief; antimicrobial activity; anti-hypertension, anti-oxidation, hepatoprotective, anti-carcinogenic and anti-inflammatory effects; and improved skeletal muscle insulin sensitivity^18^,^19^,^20^,^21^,^22^. Nevertheless, no effect on hepatic insulin resistance has been reported for DMY.

The liver is the central metabolic organ of glucose metabolism. Hepatic insulin resistance is presumed to be the primary cause of type 2 diabetes^23^. It is well established that HFD induces hallmarks of type 2 diabetes, including insulin resistance, hyperlipidemia and hepatic steatosis, in mice^24^,^25^. A HFD is also known to induce hepatic insulin resistance in mice^26^,^27^. Moreover, HepG2 cells have been shown to express various genes involved in the insulin signaling pathway and glucose metabolism via AMPK^28^,^29^. Therefore, these animal and cell models are useful tools for the analysis of glucose and lipid metabolism as well as hepatic insulin resistance^30^,^31^. In this study, rats were treated with a HFD for 8 weeks to establish an animal model of hepatic insulin resistance, and high glucose-induced HepG2 cells were used to establish a cell model, which were used to investigate the effect of DMY on glucose metabolism and insulin signaling. Therefore, the aim of this study was to determine whether dietary DMY improves hepatic glucose metabolic levels and insulin resistance in HFD-fed rats and to explore the potential molecular mechanisms of any effect.

## Materials and Methods

### Animals and Drug Treatments

Rats were randomly divided into six groups of 10 animals each. The control group that was given deionized distilled water to drink and fed standard rat chow^32^ composed of 60% vegetable starch, 12% fat, and 28% protein. The model group that was given deionized distilled water and fed a high fat diet of 60% fat, 14% protein and 26% carbohydrate. The pioglitazone group was administrated with 5 mg/kg of pioglitazone by oral gavage and fed a HFD. Pioglitazone, a potent insulin sensitizer for the treatment of type II diabetes, has an obvious effect on glucose metabolism and was used as a positive control (5 mg/kg)^33^,^34^. The rats in the DMY three groups were as follows: low dose of DMY (100 mg/kg of body weight + HFD), middle dose of DMY (200 mg/kg of body weight + HFD), high dose of DMY (400 mg/kg of body weight + HFD). (SI Materials and Methods).

The study was approved by the Ethics Committee of the Institute of Medicinal Plant Development, CAMS&PUMC (Beijing, China). All experimental procedures were performed in accordance with relevant guidelines approved by the Ethics Committee of the Institute of Medicinal Plant Development, CAMS&PUMC.

### Metabolic Measurements

Insulin tolerance testing (ITT) was performed as previously described (8) (SI Materials and Methods). All serum and hepatic biochemical parameters, except for serum insulin, were measured using an appropriate kit (Jian Cheng Biotechnology Company, Nanjing, China) according to the manufacturer’s instructions. Serum insulin levels were determined using a radioimmunoassay kit (Beijing North Institute of Biological Technology, Beijing, China) according to the manufacturer’s instructions.

### Liver Histology

Liver samples were fixed in 4% buffered neutral formalin solution for at least 24 h, then embedded in paraffin wax and sectioned (5 μm thickness) for histopathological evaluation. Liver sections were stained with H&E.

### Determination of a homeostasis model for insulin resistance (HOMA-IR)

Because abnormalities in insulin action are poorly represented by a single determination of glucose or insulin levels^35^, a homeostasis model was used to estimate insulin resistance (HOMA-IR) as follows^36^:

HOMA-IR = [Fasting insulin level (μU/ml)] × [Fasting blood glucose (mmol/l)]/22.5

### Glucose uptake and glycogen synthesis assay

Glucose uptake rates were measured after the addition of the tracer 2-NBDG to the culture medium as previously reported^37^. The accumulation of glycogen was determined using a Glycogen Colorimetric/Fluorometric Assay Kit (K646-100, BioVision, USA) as described previously^38^ (SI Materials and Methods).

### siRNA-mediated LKB1 knockdown

HepG2 cells were transfected with negative-control siRNA or LKB1 siRNA according to the manufacturer’s protocol (SI Materials and Methods).

### Metabolite profiling

Metabolite profiling was performed according to previous reports^39^ (SI Materials and Methods).

### Quantitative Real-time PCR Analysis

Total RNA was isolated from tissue, converted to complementary DNA (cDNA), and then used to measure transcripts in multiple tissues using gene-specific primers (SI Table 1), as described previously (8) (SI Materials and Methods).

### Western Blotting Assay

Total protein was isolated from livers and cells of the different experimental groups using the Protein Extraction Kit (BestBio, Shanghai, China). To assess the amount of plasma membrane-localized GLUT1, plasma membrane was isolated from HepG2 cell lysates according to the protocol of Nishiumi *et al*.^40^. The assays were performed using previously described methods^41^,^42^ (SI Materials and Methods).

### Statistical analysis

The MS raw data of cellular extracts were processed using the Thermo Xcalibur (Thermo, USA). The spectral data were analyzed as ourprevious reports^43^. The resulting data were mean-centered and pareto-scaled prior to the statistical analysis via supervised partial least-squares discriminant analysis (PLS-DA) to differentiate each group. PLS-DA was used to visualize the maximal difference of the global metabolic profiles. The data were expressed as the mean ± the standard error of the mean (SEM). The statistical analysis was performed by conducting one-way ANOVA using SPSS software v.17.0, and least-significant difference post hoc in multiple comparisons was used to examine statistical significance (p < 0.05 and p < 0.01) between groups.

## Results

### DMY improves serum glucose and lipid homeostasis and protects against HFD-induced insulin resistance

**Figure 1:**
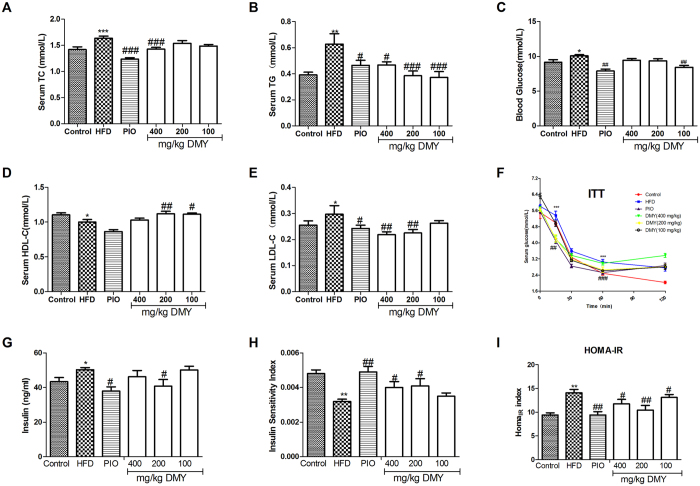
DMY partially improves serum glucose and lipid homeostasis and protects against HFD-induced insulin resistance. Serum levels of TC (**A**), TG (**B**), blood glucose (**C**), HDL-C (**D**), LDL-C (**E**) and insulin (**G**) were measured after 8 weeks of treatment. (**F**) ITT was also performed. (H) The insulin sensitivity index (ISI) was measured according to the formula ISI = 1/(fasting insulin × fasting plasma glucose). (**I**) The HOMA_IR_ index of IR was determined as follows: blood glucose (mmol/L) × serum insulin (mg/ml)/22.5. The data shown represent the means ± SEM. *P < 0.05, **P < 0.01, ***P < 0.001 compared to normal controls; ^#^*P* < 0.05, ^##^*P* < 0.01, ^###^*P* < 0.001 compared to the HFD model group, n = 10.

HFD-fed rats exhibited significantly higher serum levels of cholesterol (TC), triglyceride (TG), low-density lipoprotein cholesterol (LDL-C) and blood glucose (Fig. 1A–C,E) and lower levels of high-density lipoprotein cholesterol (HDL-C) (Fig. 1D) after 8 weeks of treatment. No significant changes were observed in lipid profiles after 4 weeks, with the exception of TC (Fig. S1). Treatment with DMY for 8 weeks significantly improved lipid profiles and reduced serum levels of glucose and insulin. The administration of DMY (100, 200 and 400 mg/kg) or PIO (5 mg/kg) significantly reduced fasting glucose and insulin levels (DMY 200 mg/kg) after as little as 4 weeks of treatment (Fig. S1). Moreover, HFD-fed rats showed a clear increase in the production of insulin and in the homeostasis model assessment-estimated IR (HOMA_IR_) index (Fig. 1G,I), while the insulin sensitivity index significantly decreased in the HFD group (Fig. 1H). Treatment with DMY (100–400 mg/kg) or PIO (5 mg/kg) alleviated the HOMA_IR_ index, consistent with an increased insulin sensitivity index after 8 weeks of treatment with DMY (200 and 400 mg/kg) or PIO (5 mg/kg) (Fig. 1H). This result was further supported by the insulin tolerance test (ITT) (Fig. 1F).

Histological examination of the livers of HFD-treated rats revealed lipid accumulation and fatty degeneration in hepatocytes (Fig. S2). However, treatment with DMY (100, 200 and 400 mg/kg) or PIO (5 mg/kg) significantly attenuated the formation of fat vacuoles in liver sections (Fig. S2). DMY or PIO also reduced hepatic TC and TG (Fig. S2) content in accordance with our pathological findings.

### DMY partially reversed the metabolic changes induced by HFD in rats

**Figure 2:**
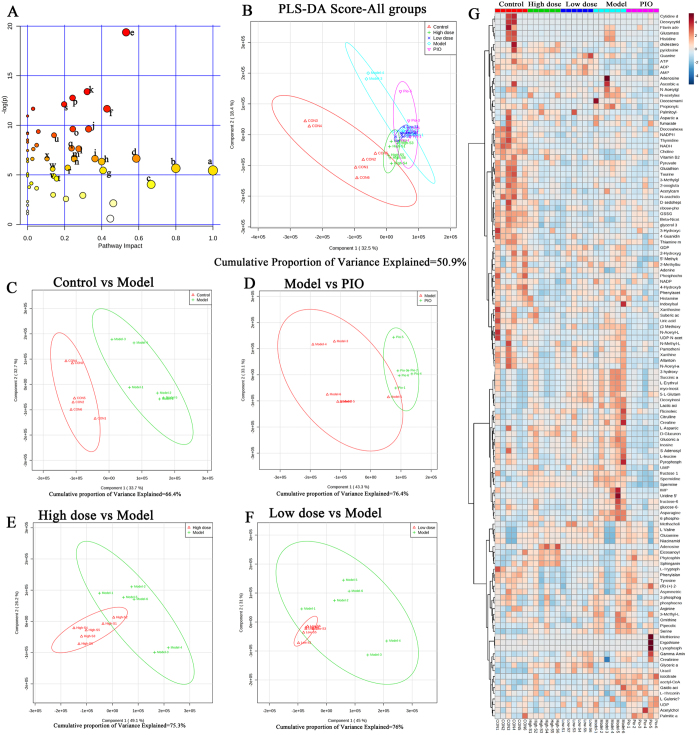
Variance analysis of metabolites based on HPLC/MS data for rat livers. (**A**) A summary of the pathway analysis using MetPA. (**B**) Plotting PLS-DA scores reveals differences in the metabolic states of the normal control group (red Δ), the 400 μmol/L DMY (high dose) group (green +), the 100 μmol/L DMY (low dose) group (blue ×), the HFD model group (brilliant blue O), and the pioglitazone (positive control) group (purple ∇). PLS-DA score plots of the HPLC/MS data for all groups. (**C**) A PLS-DA score plot showing the difference in metabolic state between the normal control group (red **Δ**) and the HFD model group (green **+**). (**D**) A PLS-DA score plot showing the difference in the metabolic state between the HFD model group (red **Δ**) and the pioglitazone (positive control) group (green **+**). (**E**) A PLS-DA score plot showing the difference in metabolic state between the 400 μmol/L DMY (high dose) group (red **Δ**) and the HFD model group (green **+**). (**F**) A PLS-DA score plot showing the difference in the metabolic state between the 100 μmol/L DMY (low dose) group (red **Δ**) and the HFD model group (green **+**). (**G**). Heat map visualization of a correlation analysis of all metabolites. Rows: samples; columns: metabolites. The color key indicates the correlation score: blue, lowest; red, highest. n = 6 rats per group. All metabolic pathways are listed in Table S2.

Changes in important positions in a network more strongly impact the pathway than changes occurring in marginal or relatively isolated positions. MetaboAnalyst 3.0 revealed that differential metabolite content is important for the normal response to the HFD, and multiple pathways are altered in HFD and DMY rats (Fig. 2A). The impact-value threshold calculated via pathway topology analysis was set to 0.10^40^, and 24 of the regulated pathways were identified as potential target pathways (Fig. 2A and Table S2) of DMY in HFD-fed rats. Figure 2B shows a two-dimensional score plot of the first two major components (PC1 and PC2), with clustering for each group. These results show a clear separation of the HFD and control groups, suggesting that severe metabolic perturbation occurs in HFD-fed rats (Fig. 2C). The DMY high dose group (400 mg/kg) and the PIO group were largely separated from the HFD group (i.e., model group); the DMY low dose group (100 mg/kg) group exhibited a small therapeutic effect and partially overlapped with the model group, suggesting that high doses of DMY and PIO ameliorated the effect on insulin resistance (Fig. 2D–F). Furthermore, unsupervised hierarchal cluster analysis revealed the fluctuation of levels across different groups, as visualized by a heat map (Fig. 2G). Metabolic substrates of different treatment groups were changed in different degrees.

### DMY ameliorates the effect on metabolic pathways induced by a HFD

**Figure 3:**
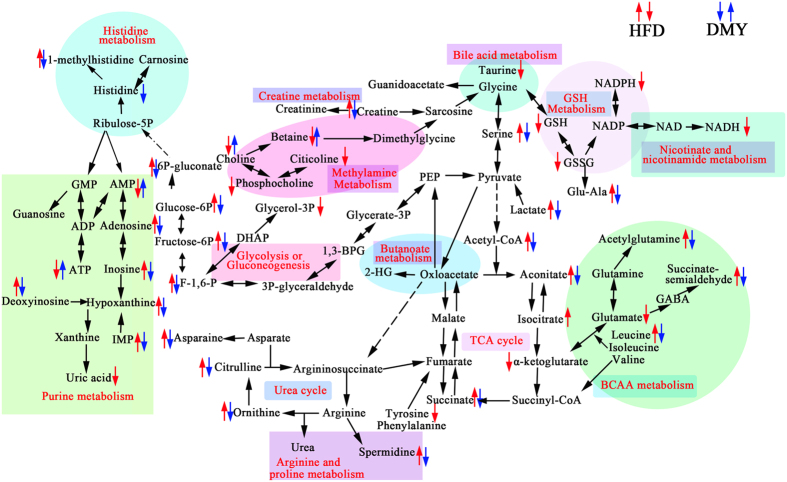
Metabolic disturbances in HFD-induced insulin-resistant rats. The metabolic pathways affected by DMY treatment are indicated by blue arrows, while those affected by HFD are indicated by red arrows. ↑, upregulated; ↓, downregulated.

The observed latent metabolites were found to be associated with purine metabolism (inosine-5′-monophosphate (IMP), hypoxanthine, uric acid, deoxyinosine, inosine, adenosine, AMP, ATP); liver injury (taurine); glycolysis (lactate, F-1,6-P, fructose-6P, glucose-6P and 6P-gluconate) and TCA cycle intermediates (acetyl-CoA, aconitate, isocitrate, α-ketoglutarate, and succinate); amino acid metabolism (acetylglutamine, succinate semialdehyde, glutamate, leucine, asparagine, serine); methylamine metabolism (betaine, phosphatidylcholine, choline, citicoline); choline metabolism (betaine); GSH metabolism (GSH, GSSG, Glu-Ala); histidine metabolism (histidine, 1-methylhistidine); arginine and proline metabolism (spermidine); the urea cycle (ornithine, citrulline), nicotinate and nicotinamide metabolism (NADH); and creatine metabolism (creatine) (Fig. 3).

### Western blot and quantitative RT-PCR analysis of key enzymes involved in the pathways of altered metabolites

**Figure 4:**
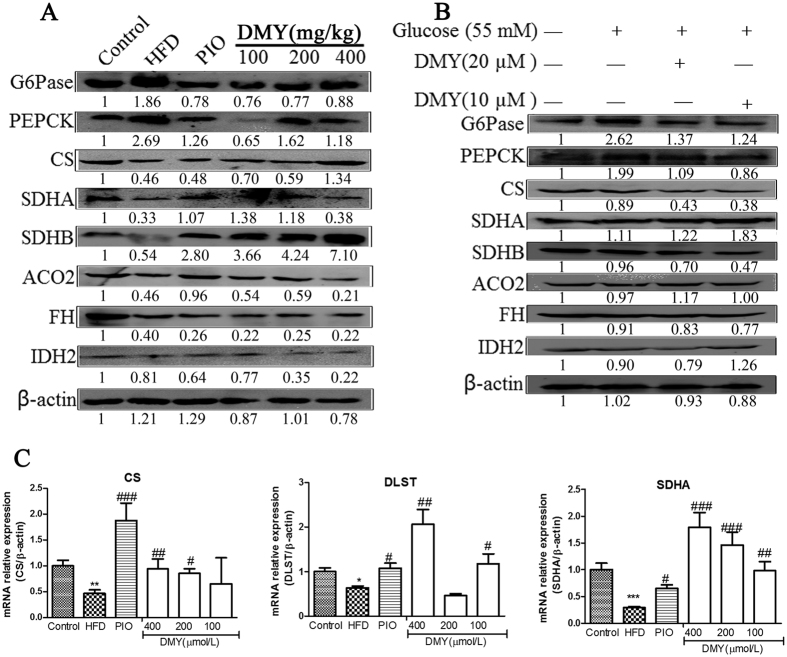
The effects of DMY on mRNA and protein levels of gluconeogenesis- or Krebs cycle-related genes *in vitro* and *in vivo*. (**A**) Western blot analysis demonstrated the effect of DMY on the expression of PEPCK, G6Pase, CS, SDHA, SDHB, ACO2, FH and IDH2 protein in HFD-fed rats. (**B**) Western blot analysis demonstrated the effect of DMY on the expression of PEPCK, G6Pase, CS, SDHA, SDHB, ACO2, FH and IDH2 protein in high glucose-induced HepG2 cells. (**C**) The effect of DMY on the mRNA expression levels of Krebs cycle genes, including CS, DLST and SDHA in HFD-fed rats (n = 4). *p < *0.05* vs the control group; **p < *0.01* vs the control group; ***p < *0.001* vs the control group; ^#^*p* < *0.05* vs the HFD model group; ^##^*p* < *0.01* vs the HFD model group.

To validate the aforementioned metabolic changes, the expression of key enzymes in these pathways, including G6Pase, PEPCK, citrate synthase (CS), succinate dehydrogenase complex, subunit A, flavoprotein (SDHA), SDHB, ACO_2_, FH and IDH_2_, was assessed at the protein and gene levels in liver tissues and HepG2 cells. As shown in Figs 4 and S10, G6Pase and PEPCK, which are involved in gluconeogenesis, showed significant upregulation in the HFD groups and in high glucose-induced HepG2 cells. Moreover, DMY treatment significantly reduced the mRNA and protein expression of G6Pase and PEPCK. In the Krebs cycle, significant downregulation of CS, SDHA, SDHB, ACO2 and FH was observed, but there was no significant difference in IDH2 between the HFD group and the control group (Fig. 4A). However, the expression of CS, SDHA, SDHB, ACO2, FH and IDH2 did not differ significantly between high glucose-induced HepG2 cells and control cells (Fig. 4B). Quantitative real-time PCR analysis revealed that HFD-fed rats exhibited a significant decrease in CS, SDHA and DLST expression, whereas this effect was inhibited by DMY administration (Fig. 4C).

### DMY stimulates glucose uptake via the Akt-GLUT1 signaling pathway

**Figure 5:**
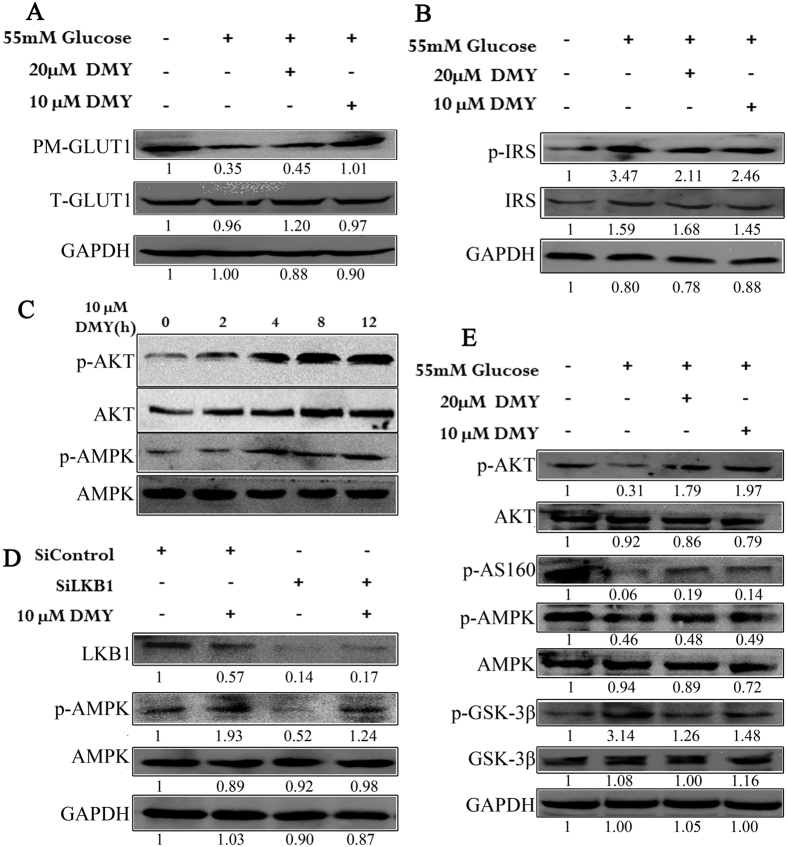
DMY activates the AMPK/GSK-3β and Akt/AS160 signaling pathways in HepG2 cells. (**A**) DMY protects GLUT1 tracking against the effects of high glucose levels in HepG2 cells. (**B**) DMY decreases the glucose-induced phosphorylation of IRS1 in HepG2 cells. (**C**) DMY stimulates the phosphorylation of Akt and AMPK in HepG2 cells. (**D**) The phosphorylation of AMPK activated by DMY is not regulated by LKB1 in HepG2 cells. (**E**) DMY modulates the AMPK/GSK-3β and Akt/AS160 signaling pathways in high glucose-induced cells.

To understand the mechanism of improved insulin sensitivity in rats treated with DMY, the protein levels of key mediators of insulin signaling cascades were examined in the high glucose-induced HepG2 insulin resistance model. The involvement of DMY in insulin signaling pathways was also examined. As shown in Fig. 5A, the level of GLUT1 in the plasma membrane was significantly decreased by 55 mmol/L glucose and was increased by pretreatment with DMY (10 μmol/L), whereas there was no significant difference the level of GLUT1 in the cytoplasm. The phosphorylation of IRS-1 at Ser 612 was downregulated in HepG2 cells pretreated with DMY compared with the high glucose group (Fig. 5B). The phosphorylation of Akt at Ser474 and AMPK at 172 was significantly higher in cells pretreated with DMY (10 μmol/L) for 4–12 h than in the high glucose group (Fig. 5C). LKB1-specific siRNA was introduced into HepG2 cells to evaluate the role of LKB1 in DMY activity (Fig. 5D). siRNA-mediated knockdown of LKB1 did not abolish the increase in the phosphorylation of AMPK induced by DMY. Western blot analyses revealed that the phosphorylation of Akt, AS160 and AMPK were significantly decreased in high glucose-induced HepG2 cells compared to the control group. DMY significantly blunted these decreases (Fig. 5E); compared with the control group, high glucose-induced HepG2 cells displayed increases in the levels of phosphorylated GSK-3β protein by Western blot analysis. The administration of DMY normalized the high glucose-induced increases in phosphorylated GSK-3β protein levels.

## Discussion

The research findings showed that the HFD induced marked insulin resistance, as demonstrated by elevated blood glucose; insulin secretion; and impairments in insulin sensitivity, IRS/Akt-GLUT2 signaling, glycolysis, Krebs cycle, and gluconeogenesis. Interestingly, we found that oral administration of DMY improved hepatic insulin resistance and significantly restored glucose metabolic homeostasis in HFD-fed rats. Therefore, we speculate that DMY plays a protective role in HFD-induced hepatic insulin resistance, and we performed additional experiments to investigate this effect.

HFD feeding in rats results in fat accumulation in the liver as well as insulin resistance, pathophysiologic features that are analogous to those of human clinical metabolic disease. Indeed, our observations demonstrate obvious fat accumulation in the liver after feeding a HFD at both 4 weeks and 8 weeks, as evidenced by increases in plasma TG and TC (Figs S1 and 1), hepatic TC and TG (Fig. S2), and HE staining (Fig. S2). These phenotypic alterations were clearly antagonized by DMY treatment. Moreover, DMY treatment improved blood glucose, serum HDL-C, serum LDL-C, and the insulin sensitively index induced by HFD feeding and glucose uptake in HepG2 cells or high glucose-induced HepG2 cells (Figs 1 and S9). Taken together, our results demonstrate that DMY has the ability to inhibit hepatic fat accumulation, increase glucose uptake, and improve insulin resistance.

The liver is a main site of glucose metabolism and is stimulated by insulin to import glucose from the blood and synthesize glycogen, thereby preventing postprandial hyperglycemia^44^,^45^. Under insulin-resistant conditions, hepatic gluconeogenesis is elevated^7^, and the key gluconeogenic enzymes G6Pase and PEPCK are significantly increased in the livers of HFD-fed mice^46^. In this study, HFD feeding and high glucose stimulation indeed promoted gluconeogenesis, as shown by an increase in the expression of G6Pase and PEPCK (Fig. 4). Lactate, G-3-P, and F-6-P were correlated with glycolysis, suggesting modulation of the glycolytic pathway. A reduction in lactate levels was observed in the HFD group, which suggests a decreased rate of glycolysis and increased the expression of glycolysis-related enzymes in HFD-fed rats (Figs S3 and S11), consistent with a previous study^47^. Intriguingly, DMY treatment reversed these pathological changes (i.e., the reduction in the expression of G6Pase and PEPCK and the increase in the level of lactate). These results demonstrate that DMY treatment exerts an effect on HFD-fed rats via an improvement in gluconeogenesis and glycolysis.

The tricarboxylic acid (TCA) cycle results in terminal fat oxidation and is a metabolic precursor of gluconeogenesis. TCA intermediates and the components of glucose metabolism are strongly associated; insulin resistance promotes hepatic TCA flux in mice tending toward insulin resistance due to a HFD^48^. Indeed, our LC-MS metabolomic data revealed five pivotal intermediates of the TCA cycle in the present study; low levels of 2-oxoglutarate and high levels of acetyl-CoA, cis-aconitate, isocitrate, and succinate were observed in HFD-fed rats (Fig. S3). These results are in line with the reported higher levels of citrate in T2DM rhesus macaques compared with normal animals^49^. In addition, Sprague-Dawley rats with T1DM induced by streptozotocin exhibited higher levels of pyruvate, succinate and fumarate^50^. The metabolism of succinate, acetyl-CoA and citrate depends on succinate dehydrogenase (SDH) and citrate synthetase (CS), while the metabolism of 2-oxoglutarate depends on dihydrolipoyl succinyltransferase (DLST). Real-time PCR and Western blot analysis revealed that CS, SDHA and DLST (by qPCR only) were downregulated in the HFD group, which likely contributed to the observed changes in succinate, acetyl-CoA, citrate and 2-oxoglutarate (Fig. 4C). Our results indicate by an array of mechanisms that DMY contributes to reducing blood glucose, particularly by regulating the TCA cycle pathway and decreasing hepatic gluconeogenesis.

Most amino acid metabolism occurs in the liver, and a broad range of glucogenic amino acids are used for hepatic gluconeogenesis^51^. DMY reversed the elevated levels of 5-L-glutamyl-alanine, L-methylhistidine, SSA, asparagine, serine and leucine (Figs S4 and S5) observed in HFD-fed rats. However, lower levels of tryptophan and glucogenic amino acids (i.e., tyrosine and phenylalanine) were observed in HFD-fed rats compared with the normal group (Fig. S4). Our results are consistent with those of a previous study, wherein a HFD caused the impairment of insulin signaling and the regulation of gluconeogenesis^52^. The reduction in glucogenic amino acids may reflect the promotion of gluconeogenesis, which is observed when there is an increased level of glucose (Figs 1 and 4). In the liver, the enhanced use of amino acids for gluconeogenesis is consistent with the observed increase in urea cycle-related metabolites (citrulline) as insulin resistance develops (Fig. S4). Previous studies have reported the combined elevation of taurine and creatine levels as a biomarker for liver damage^53^. This decrease in liver taurine in HFD-fed rats is the result of leakage from damaged hepatocytes into urine and the inhibition of protein synthesis by hepatotoxicants, which has been shown to increase urinary taurine excretion in rats^54^,^55^. The increased creatine and diminished taurine levels in HFD-fed rats are consistent with the aforementioned reports (Fig. S4). Our data demonstrate that DMY treatment results in the normalization of these effects, suggesting that DMY could inhibit HFD-induced liver damage. Betaine is an essential osmoregulatory compound and an important cofactor of methylation during the methionine-homo-cysteine cycle^56^. Previous work has shown that betaine insufficiency is associated with metabolic syndrome, lipid disorders and diabetes^57^. Moreover, betaine administration was found to significantly improve insulin resistance in a HFD-fed animal model^58^. In the current study, compared with the normal diet group, a decreased level of betaine was observed in the HFD-fed rats (Fig. S6). Our analysis shows that the level of betaine in DMY-treated rats recovered to those of control rats. Therefore, we suggest that the ethylamine metabolism pathway could be another treatment target of DMY. In addition, we found that DMY-treated rats trended towards normal for many metabolic substrates, including D-glucuronic acid, gluconic acid, 3-hydroxy-3-methyl-glutaric acid, 3-hydroxycaproic acid, pipecolic acid, pyridoxine-5′-phosphate, vitamin B2, and docosenamide (Figs S6 and S7).

Previous studies have reported profound changes in metabolic substrates of purine and pyrimidine metabolism in diabetic or insulin-resistant rats^59^,^60^. Our results also indicate that the breakdown of pyrimidines proceeds in parallel with that of purines and that the metabolism of purine and pyrimidine nucleotides during diabetes is codependent (Fig. S8). In this study, we also observed a significant increase in uridine concentration in the liver tissues of HFD-fed rats (Fig. S8). Compared with HFD-fed rats, the DMY groups exhibited similar levels of the metabolic substrates of purines and pyrimidines.

We further investigated which signaling pathways are involved in DMY-induced changes in liver energy metabolism. It is known that insulin is a major regulator of glucose transport via the receptor-guided intracellular IR substrate-1 (IRS-1)/PI3K/Akt pathway^61^. In this study, DMY downregulated p-IRS (ser612) and upregulated p-Akt in high glucose-induced insulin resistant cells (Fig. 5B,E). Insulin/Akt/FOXO1 represents another important signaling axis that controls hepatic glucose production and metabolism^62^. DMY could ameliorate the HFD- or high glucose-impaired phosphorylation of Akt and increased expression of G6pase and PEPCK (Fig. 5). In turn, this results in chronic hyperglycemia and widespread oxidative stress as well as whole-body insulin resistance under regular feeding conditions^62^. In addition, DMY inhibited the high glucose-impaired phosphorylation of Akt, resulting in the hypophosphorylation of GSK-3β (Fig. 5E), which may contribute to the development of insulin resistance in rodents and humans^63^. Many studies have shown that HFD significantly decreases hepatic AMPK and Akt phosphorylation levels, thereby inhibiting the translocation of GLUT2 to the plasma membrane and resulting in hepatic insulin resistance^64^,^65^,^66^. Moreover, our research showed that DMY stimulated the phosphorylation of AMPK and AS160. This could promote GLUT1 translocation (Fig. 5A,E) because AMPK acts as a glucose sensor and contributes to increased insulin-independent glucose uptake and the maintenance of glucose homeostasis^67^. Thus, the positive effects of DMY on insulin resistance may be mediated, at least in part, by the activation of the adiponectin-AMPK signaling pathway. However, no studies have defined the upstream signaling pathways involved in flavanone-induced AMPK activation. In the current study, we found that DMY-induced AMPK phosphorylation was not blocked by LKB1 siRNA in HepG2 cells (Fig. 5D). This finding clearly suggests that DMY activates the AMPK signaling pathway but not the LKB1/AMPK pathway.

In conclusion, the present study demonstrates that DMY reduces hepatic insulin resistance in HFD-fed rats. Our results suggest that the targets of DMY treatment are glucose metabolism (glycolysis and gluconeogenesis), the TCA cycle, amino acid metabolism, and purine and pyrimidine metabolism. Our findings also provide physiological and molecular evidence that DMY improves insulin resistance in HFD-fed rats by (i) stimulating GLUT1 translocation from the cytosol to the membrane through the activation of AMPK signaling to promote glucose uptake, and (ii) regulating the expression of G6Pase and PEPCK via the IRS/PI3K/Akt pathways to decrease glucose production. Given the strong clinical interest in developing novel pharmacological agents that can mitigate both hyperglycemia and the consequences of liver fat a ccumulation, our data demonstrate that DMY possesses both of these properties and therefore should be developed as a novel agent to improve type 2 diabetes insulin resistance.

## Additional Information

**How to cite this article**: Le, L. *et al*. Metabolomics reveals the protective of Dihydromyricetin on glucose homeostasis by enhancing insulin sensitivity. *Sci. Rep.* **6**, 36184; doi: 10.1038/srep36184 (2016).

**Publisher’s note:** Springer Nature remains neutral with regard to jurisdictional claims in published maps and institutional affiliations.

## Supplementary Material

Supplementary Information
