# Supplementary material for: Metabolomics reveals the protective of Dihydromyricetin on glucose homeostasis by enhancing insulin sensitivity
Source: Sci Rep. 2016 Oct 31;6:36184. doi: 10.1038/srep36184 (PMC5087077; doi:10.1038/srep36184)
Supplement: Supplementary Information [file srep36184-s1.pdf]

Metabolomics reveals the protective of Dihydromyricetin on glucose homeostasis by enhancing insulin sensitivity

Liang Le[1#], Baoping Jiang[1,2#], Wenting Wan[1,2], Wei Zhai[1], Keping Hu[*1], Lijia Xu[1,2*], Peigen Xiao[1,2*]

[1] Institute of Medicinal Plant Development, Chinese Academy of Medical Sciences, Peking Union Medical College, Beijing, 100193

[2] State Key Laboratory of Bioactive Substances and Resources Utilization of Chinese Herbal Medicine, Ministry of Education, Beijing

*Correspondence to: Lijia Xu, Institute of Medicinal Plant, Peking Union Medical College, Chinese Academy of Medical Sciences. No 151, North Road Malianwa, Haidian District, Beijing 100193, China

Tel: +86-01057833153; fax: +86-010-57833165. Email: xulijia@hotmail.com

*Correspondence to: Keping Hu, Institute of Medicinal Plant, Peking Union Medical College, Chinese Academy of Medical Sciences. No 151, North Road Malianwa, Haidian District, Beijing 100193, China. Email: hukeping77@gmail.com

*Correspondence to: Peigen Xiao, Institute of Medicinal Plant, Peking Union Medical College, Chinese Academy of Medical Sciences. No 151, North Road Malianwa, Haidian District, Beijing 100193, China

Tel: +86-010-63011294; fax: +86-010-57833165. Email: pgxiao@implad.ac.cn

# These authors contributed equally to this work

SUPPLEMENTARY METHODS

Materials

HepG2 cells were purchased from the Cell Bank of the Chinese Academy of Sciences (Beijing). Dulbecco's modified Eagle's medium (DMEM), fetal bovine serum (FBS), and other tissue culture reagents were purchased from Gibco (Life Technologies, USA). 2-(N-(7-Nitrobenz-2-oxa-1, 3-diazol-4-yl) Amino)-2-Deoxyglucose (2-NBDG) and LKB1 siRNA oligonucleotides (siRNA ID; s13581) were purchased from Life Technologies Co. (USA). Other reagents were purchased from Sigma-Aldrich Co. (St. Louis, MO, USA) unless otherwise indicated. Dihydromyricetin (DMY), with a purity of 97% based on reversed-phase HPLC analysis, was prepared by our group as previously reported [1].

Animals and diets

Male Sprague-Dawley (SD) rats weighing 100-140 g were kept in a humidity-controlled and air-conditioned room (22±2°C) with a 12 h light cycle (6:00 a.m.-6:00 p.m.) and food and water available *ad libitum* for 1 week. The rats were divided randomly into six groups of 10 animals each: a control group that was given deionized distilled water to drink and was fed standard rat chow [2] composed of 60% vegetable starch, 12% fat, and 28% protein; and a model group that was given deionized distilled water and fed a diet of 60% fat, 14% protein and 26% carbohydrate. The rats in the DMY groups were treated with 100, 200, and 400 mg/kg DMY by oral gavage. Pioglitazone, a potent insulin sensitizer used in the treatment of type II diabetes, has a known effect on glucose metabolism and was used as a positive control (5 mg/kg) [3,4]. During the 8-week study period, fresh water or flower tea was provided daily at 7:00 PM. After treatment for 4 weeks, blood samples were collected from the ocular vein in heparinized tubes after overnight fasting, and the plasma was separated by centrifugation and stored at -20°C until subsequent use. After blood sampling, the animals were sacrificed following anesthetization by chloral hydrates (350 mg/kg, i.p.) at the end of the 8-week period. The livers were removed at the time of death and immediately frozen in liquid nitrogen for Western blot or PCR analysis. All serum and hepatic biochemical parameters, with the exception of serum insulin, were measured by the respective kits (Jian Cheng Biotechnology Company, Nanjing, China) according to the manufacturer's instructions. Serum insulin levels were determined using a radioimmunoassay kit (Beijing North Institute of Biological Technology, Beijing, China) according to the manufacturer's instructions.

The study was approved by the Ethics Committee of the Institute of Medicinal Plant Development, CAMS&PUMC (Beijing, China). All experimental procedures were performed in accordance with relevant guidelines approved by the Ethics Committee of the Institute of Medicinal Plant Development, CAMS&PUMC.

Histological analysis

Liver tissue specimens were fixed in a 4% buffered neutral formalin solution for at least 24 h,

embedded in paraffin wax and sectioned (5 μm thickness) for histopathological evaluation. Liver sections were stained with H&E. The images were observed under a light microscope and photographed at 400× final magnification.

Measurement of insulin tolerance

During the last week of treatment and after a 12 h fasting, the animals were orally gavaged with 2 g/kg body weight glucose dissolved in water, then injected subcutaneously with insulin (0.75 U/kg body weight). Blood samples were taken from the tail vein at 0, 15, 30, 45, 60, 90 and 120 min after glucose loading, and plasma glucose levels were assessed.

Homeostasis model of insulin resistance (HOMA-IR)

Because abnormalities in insulin action are poorly represented by a single measurement of glucose or insulin levels [5], a homeostasis model was used to estimate insulin resistance (HOMA-IR) as follows [6]:

HOMA-IR=[Fasting insulin level (μU/ml)] × [Fasting blood glucose (mmol/l)]/22.5

Cell culture

Human HepG2 cells were cultured in low-glucose DMEM (5.5 mM glucose) supplemented with 10% FBS and 1% antibiotics and incubated at 37°C in humidified air containing 5% CO_2. Cells were grown to 70-80% confluence and then incubated in 2% FBS in DMEM overnight. Cells were washed and incubated with the indicated concentration of the respective chemical in 2% FBS/DMEM or 2% FBS/DMEM alone for the indicated amount of time. Then, the cells were cultured in growth medium containing 5 mmol/L glucose (representing normal glycemia) or 55 mM glucose (hyperglycemia (HG) and DMY groups). HepG2 cells were incubated in insulin (10^{-7}mol/L) for 15 min before initiation of the various treatments.

Glucose uptake assay

Glucose uptake rates were measured after the addition of the tracer 2-NBDG to the culture medium as previously reported [7]. Cells were cultured in black 96-well plates to 90% confluence and incubated with the respective treatment then washed twice and incubated with 100 μmol/L 2-NBDG in glucose-free culture medium for 20 min. Cells cultured in the absence of 2-NBDG served as a negative control. The cells were washed twice, and fluorescence was detected using a microplate reader (Infinite 1000 M, Tecan, AUSTRIA) with excitation at 488 nm and emission at 520 nm.

Assay of glycogen synthesis

The accumulation of glycogen was determined using a Glycogen Colorimetric/Fluorometric Assay Kit (K646-100, BioVision, USA) as described previously [8]. After the conclusion of experimental treatments, the cells were washed three times with ice-cold PBS. The 106 cells were homogenized in 200 μl dH_2O on ice and then boiled for 10 min to inactivate any enzymes. The boiled samples were centrifuged at 18,000×g for 10 min to remove insoluble material, and the supernatant was assayed using a microplate reader (Infinite 1000 M, Tecan, AUSTRIA) with excitation at 488 nm and emission at 520 nm.

siRNA-mediated LKB1 knockdown

HepG2 cells were transfected with negative-control siRNA or LKB1 siRNA according to the manufacturer's protocol. Briefly, cells were seeded into 60-mm dishes. After 24 h, the medium was changed to fresh, antibiotic-free medium, and the cells were cultured for an additional 24 h in the presence or absence of DMY. siRNA plasmids were transfected into HepG2 cells using Lipofectamine 2000 (Invitrogen, Carlsbad, CA, USA) according to the manufacturer's instructions. The cells were then allowed to express the siRNA for 48 h.

Metabolite profiling

Metabolite profiling was performed according to previous reports [9]. Briefly, rat livers were harvested and lysed in a solution of methanol:chloroform:water (1:2:1) to extract metabolites. All reagents were of chromatographic grade. Metabolite profiling was performed using a TSQ Quantum Access™ triple quadrupole mass spectrometer with an electrospray ionization (ESI) source coupled to a Surveyor auto-sampler, a Surveyor LC pump, and Xcalibur3.0 software for data acquisition and analysis (Thermo Finigan, USA). Mass spectrometry was controlled by XCalibur software Version 3.0 (Thermo Electron Corporation, USA) and was operated in selected reaction monitoring (SRM) mode using electrospray ionization in the negative-ion mode. We used a specific sample as quality control (QC) rather than an internal standard. QC runs were performed at the beginning and end of the sequence. The total ion current and chromatographic patterns were evaluated. Table X shows four representative compounds identified in QC runs.

Quantitative real-time PCR analysis

Total RNA was extracted from liver tissues using the *EasyPurfe*™ RNA Kit (ER101, TransGen Biotech, Beijing China) according to the manufacturer's instructions. cDNA was synthesized using *EasyScript®* First-strand Synthesis SuperMix and a PCR System (Eppendorf AG, Germany). The assay was performed according to real-time PCR with TransStart® Top Green qPCR SuperMix (Tli RNaseH Plus) on an iCycler iQ5 thermocycler (BioRad, Hercules, CA) as previously described [10]. The primers used to amplify each gene are listed in Table 1. All samples were analyzed in triplicate, and gene expression levels were normalized to control rat β-actin values. Fold changes between the groups were calculated using the $2^{-\Delta\Delta Ct}$ method [11].

For Western blotting, total protein was isolated from the livers and cells of different treatment groups using a Protein Extraction Kit. To detect the amount of plasma membrane-localized GLUT2, the plasma membrane was isolated from HepG2 cell lysates according to the protocol of Nishiumi et al. [12]. The assays were performed using standard methods [13,14], and the membrane was incubated overnight at 4°C with the following primary antibodies: phospho-(Thr^{172})-AMPKα2 (p-AMPK), AMPKα2 (AMPK), GSK-3β, phosphor-GSK-3β (Ser9), phosphor-AS160 (Thr642), IRS-1 and phospho-(Ser^{612})-IRS-1(p-IRS-1) were purchased from Cell Signalling Technology, Inc. (USA); antibodies against Akt2, GLUT2, phospho-Akt (Ser^{474}), anti-MDH2, anti-SDHA, anti-CS and GAPDH were purchased from Abcam (UK).

SUPPLEMENTARY REFERENCES

1. Jiang B, Le L, Pan H, Hu K, Xu L, Xiao P. Dihydromyricetin ameliorates the oxidative stress

response induced by methylglyoxal via the AMPK/GLUT4 signaling pathway in PC12 cells. Brain Res Bull 2014; 109: 117-126.
2. Iizuka K, Bruick RK, Liang G, Horton JD, Uyeda K. Deficiency of carbohydrate response element-binding protein (ChREBP) reduces lipogenesis as well as glycolysis. Proc Natl Acad Sci U S A 2004; 101: 7281-7286.
3. Zhang L, Xu J, Song H, Yao Z, Ji G. Extracts from Salvia-Nelumbinis naturalis alleviate hepatosteatosis via improving hepatic insulin sensitivity. J Transl Med 2014; 12: 236.
4. Takahashi T, Yamamoto M, Amikura K *et al.* A novel MitoNEET ligand, TT01001, improves diabetes and ameliorates mitochondrial function in db/db mice. J Pharmacol Exp Ther 2015; 352: 338-345.
5. Laakso M. How good a marker is insulin level for insulin resistance? . Am J Epidemiol 1993; 137: 959-965.
6. Haffner SM, Greenberg AS, Weston WM, Chen H, Williams K, Freed MI. Effect of rosiglitazone treatment on nontraditional markers of cardiovascular disease in patients with type 2 diabetes mellitus. Circulation 2002; 106: 679-684.
7. Yoshioka K, Saito M, Oh KB *et al.* Intracellular fate of 2-NBDG, a fluorescent probe for glucose uptake activity, in Escherichia coli cells. Biosci Biotechnol Biochem 1996; 60: 1899-1901.
8. Bueding E, Orrell SA. A mild procedure for the isolation of polydisperse glycogen from animal tissues. J Biol Chem 1964; 239: 4018-4020.
9. Yuan M, Breitkopf SB, Yang X, Asara JM. A positive/negative ion-switching, targeted mass spectrometry-based metabolomics platform for bodily fluids, cells, and fresh and fixed tissue. Nat Protoc 2012; 7: 872-881.
10. MacDonald MJ, Brown LJ, Longacre MJ, Stoker SW, Kendrick MA, Hasan NM. Knockdown of both mitochondrial isocitrate dehydrogenase enzymes in pancreatic beta cells inhibits insulin secretion. Biochim Biophys Acta 2013; 1830: 5104-5111.
11. Livak KJ, Schmittgen TD. Analysis of relative gene expression data using real-time quantitative PCR and the 2(-Delta Delta C(T)) Method. Methods 2001; 25: 402-408.
12. Nishiumi S, Ashida H. Rapid preparation of a plasma membrane fraction from adipocytes and muscle cells: application to detection of translocated glucose transporter 4 on the plasma membrane. Biosci Biotechnol Biochem 2007; 71: 2343-2346.
13. Liu Y, Lu B, Peng J. Hepatoprotective activity of the total flavonoids from Rosa laevigata Michx fruit in mice treated by paracetamol. . Food Chem Toxicol 2011; 125: 719-725.
14. Lu B, Xu Y, Xu L *et al.* Mechanism investigation of dioscin against CCl4-induced acute liver damage in mice. Environ Toxicol Pharmacol 2012; 34: 127-135.

Table S1 Primer sequences used for real-time PCR

Gen	Forward/Reverse primer (5'-3')	GenBank accession
GAPDH-F	CCTTCCGTGTTCCTACCCC	NM_017008.3
GAPDH-R	GCCTGCTTCACCTTCTT	
DLST-F	GAAATAGGCTTCATGTCGG	XM_006240306.1
DLST-R	CACCTCCTTGGTTGCGTC	
SDHA-F	GTCCATACACCGAATAAGAGCA	NM_130428.1
SDHA-R	GAGGCAGCCAGCACCATA	

CS-F	GCCCTCAACAGTGAAAGCA	NM_130755.1
CS-R	TGGCAATCAGGTCCATACAG	

Table S2 Result from Metabolic Pathway Analysis with MetaboAnalyst 3.0[a]

NO.	Pathway name	Total Cmpd	Hits	Raw p	-LOG(P)	Holm adjust	FDR	Impact
a	Phenylalanine, tyrosine and tryptophan biosynthesis	4	2	0.004	5.448	0.082	0.006	1.000
b	Ascorbate and aldarate metabolism	9	4	0.003	5.664	0.073	0.006	0.800
c	Valine, leucine and isoleucine biosynthesis	11	3	0.018	4.030	0.213	0.023	0.667
d	Alanine, aspartate and glutamate metabolism	24	11	0.001	6.654	0.040	0.003	0.586
e	Glutathione metabolism	26	8	0.000	19.379	0.000	0.000	0.531
f	Taurine and hypotaurine metabolism	8	1	0.000	11.668	0.000	0.000	0.429
g	Phenylalanine metabolism	9	2	0.004	5.448	0.082	0.006	0.407
h	Methane metabolism	9	1	0.002	6.340	0.042	0.003	0.400
i	Pentose and glucuronate interconversions	14	2	0.001	6.635	0.040	0.003	0.364
j	Pyruvate metabolism	22	4	0.000	9.625	0.003	0.000	0.329
k	Purine metabolism	68	15	0.000	13.396	0.000	0.000	0.321
l	Pyrimidine metabolism	41	7	0.000	7.632	0.017	0.001	0.276
m	Citrate cycle (TCA cycle)	20	7	0.001	6.707	0.039	0.003	0.252
n	Fatty acid elongation in mitochondria	27	1	0.001	6.605	0.040	0.003	0.252
o	Glycolysis or Gluconeogenesis	26	4	0.000	9.625	0.003	0.000	0.244
p	Glycine, serine and threonine metabolism	32	7	0.000	12.764	0.000	0.000	0.243
q	Glycerolipid metabolism	18	3	0.000	7.683	0.017	0.001	0.237
r	Cysteine and methionine metabolism	28	5	0.003	5.712	0.073	0.006	0.217
s	Glycerophospholipid metabolism	30	5	0.000	12.120	0.000	0.000	0.197
t	Tryptophan metabolism	41	2	0.010	4.619	0.138	0.013	0.157
u	Sphingolipid metabolism	21	3	0.000	9.000	0.005	0.000	0.143
v	Tyrosine metabolism	42	2	0.008	4.881	0.121	0.010	0.140
w	Inositol phosphate metabolism	26	4	0.004	5.600	0.074	0.006	0.135
x	Starch and sucrose metabolism	23	2	0.001	6.635	0.040	0.003	0.105

[a]Total is the total number of compounds in the pathway; the hits is the actually matched number from the user uploaded data; the Raw p is the original p-value calculated from the enrichment analysis; FDR (False Discovery Rate) is also from the enrichment analysis; the impact is the pathway impact value calculated from pathway topology analysis.

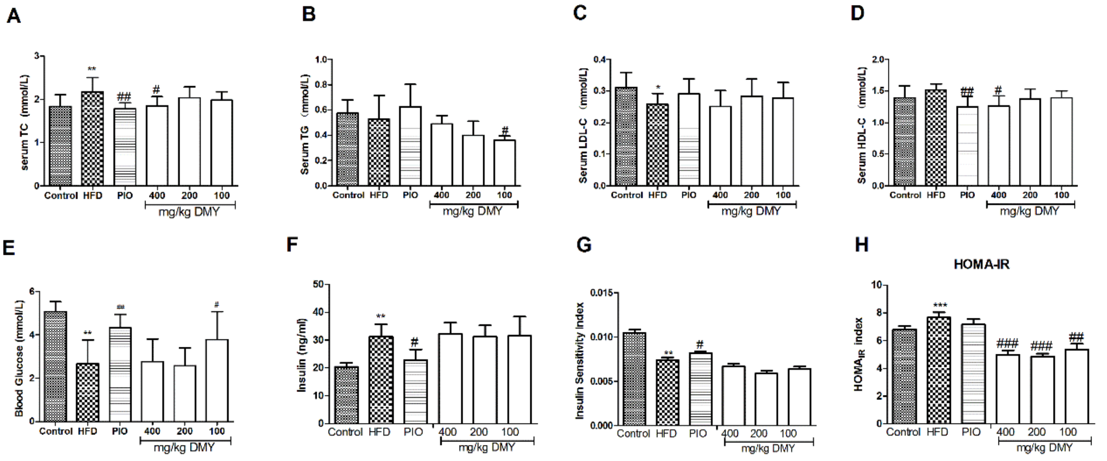

Figure S1. Effects of DMY on serum glucose and lipid profiles in HFD-treated rats for 4 weeks. The serum levels of TC (A), TG (B), LDL-C (C), HDL-C (D), glucose (E), and insulin (F) were measured at the end of 4-week treatment. (G) ITT were also performed on the animals. (H) IHOMA$_{IR}$ index of IR was determined as follows: blood glucose (mmol/L)/ serum insulin (mg/ml)/22.5. Data shown represent the means±SEM. $^{*}P$<0.05, $^{**}P$<0.01, $^{***}P$<0.001 compared to normal control; $^{\#}P$<0.05, $^{\#\#}P$<0.01, $^{\#\#\#}P$<0.001 compared to HFD model group, n=10.

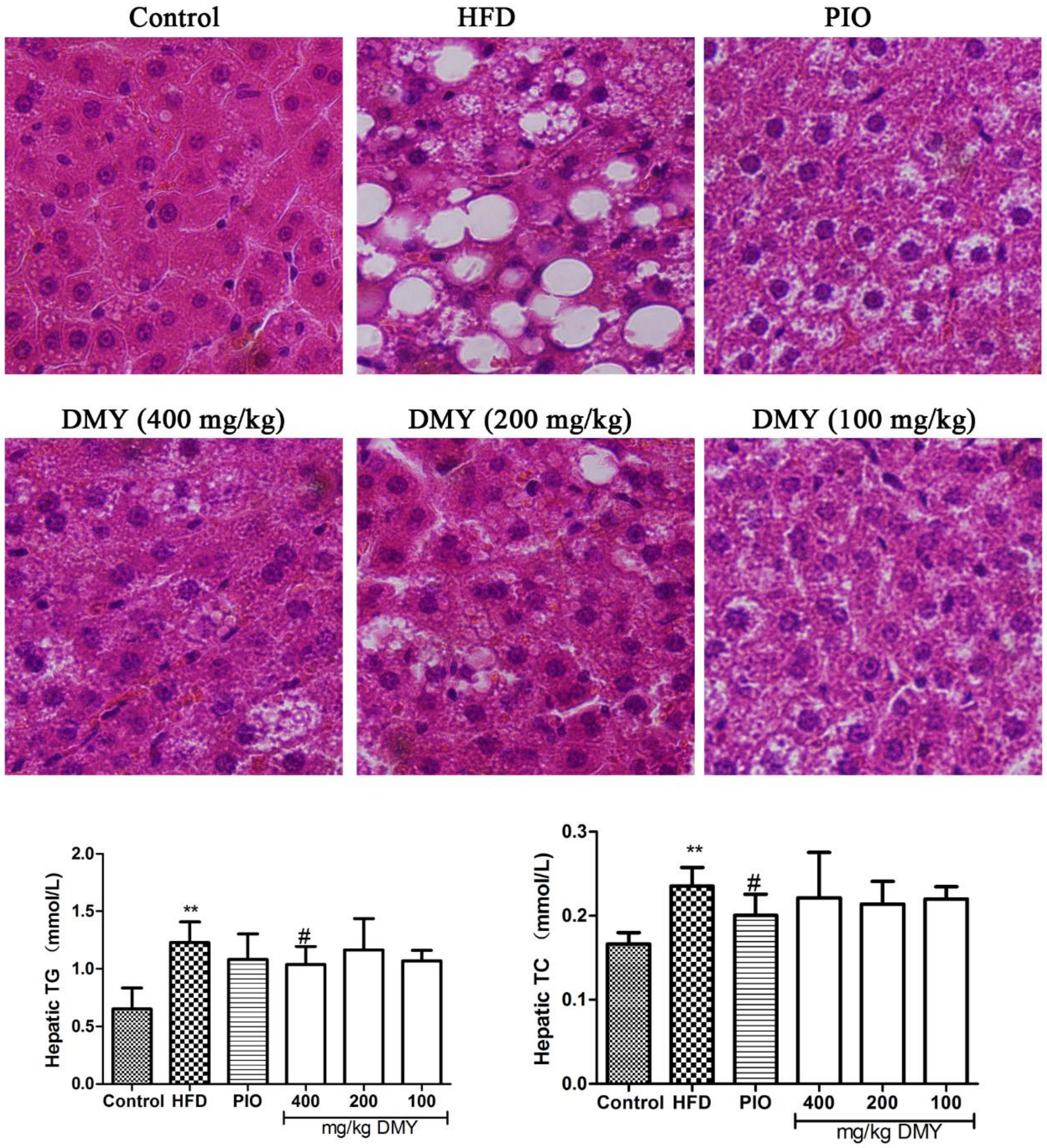

Figure S2. Effects of DMY on lipid accumulation and steatosis in HFD-treated rats for 8 weeks. Representative hematoxylin and eosin staining of the liver (magnifications of 40×). Effects of DMY on hepatic TC and TG in HFD-treated rats for 8 weeks. $^{**}p$<0.01 compared to normal control; $^{\#}p$<0.05 compared to the HFD model group, n=10.

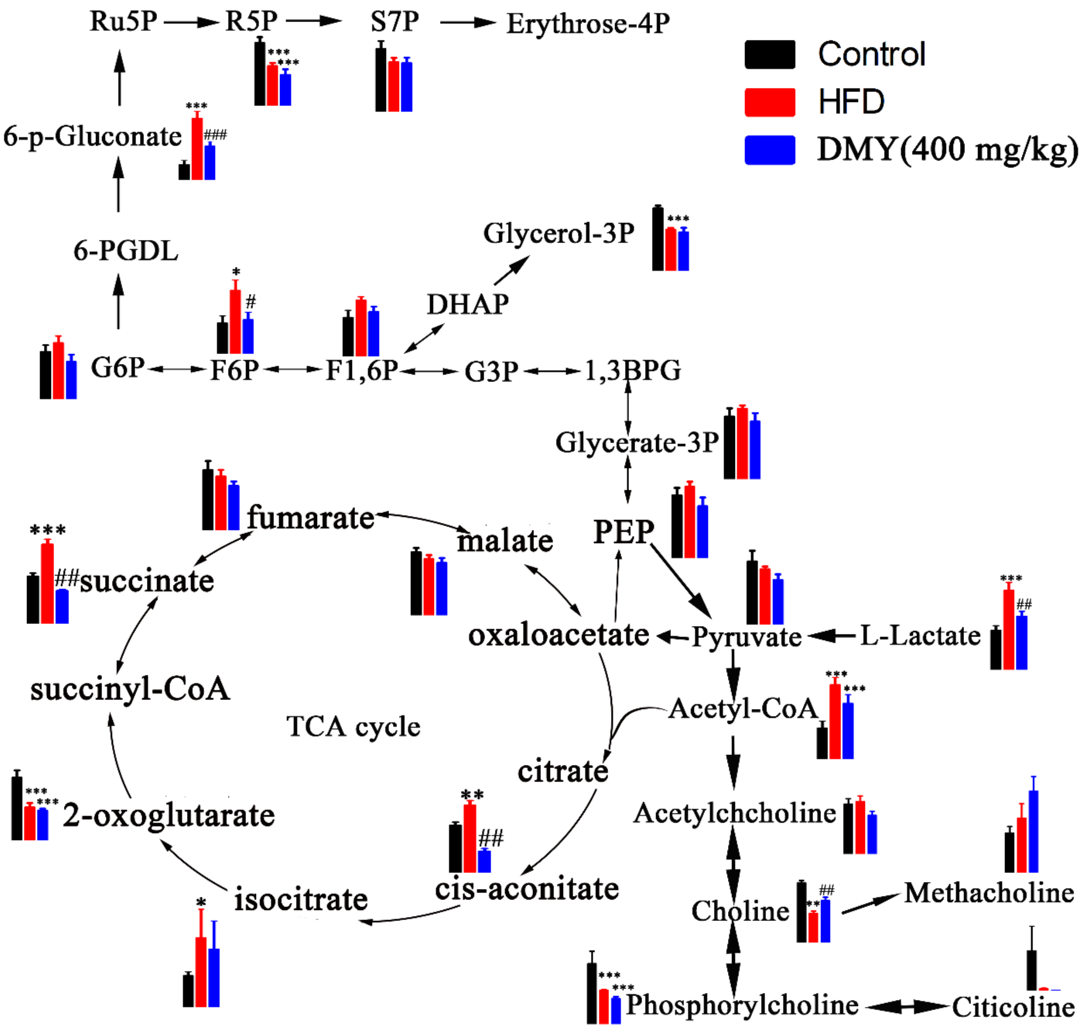

Figure S3. Metabolome pathway map of quantified metabolites, including components of the Krebs cycle, glycolysis pathway in all groups. * *P<0.05* vs the control group; ** *P<0.01* vs the control group; *** *P<0.001* vs the control group; # *P<0.05* vs HFD model group; ## *P<0.01* vs HFD model group; ###*P<0.001* vs HFD model group, n=6.

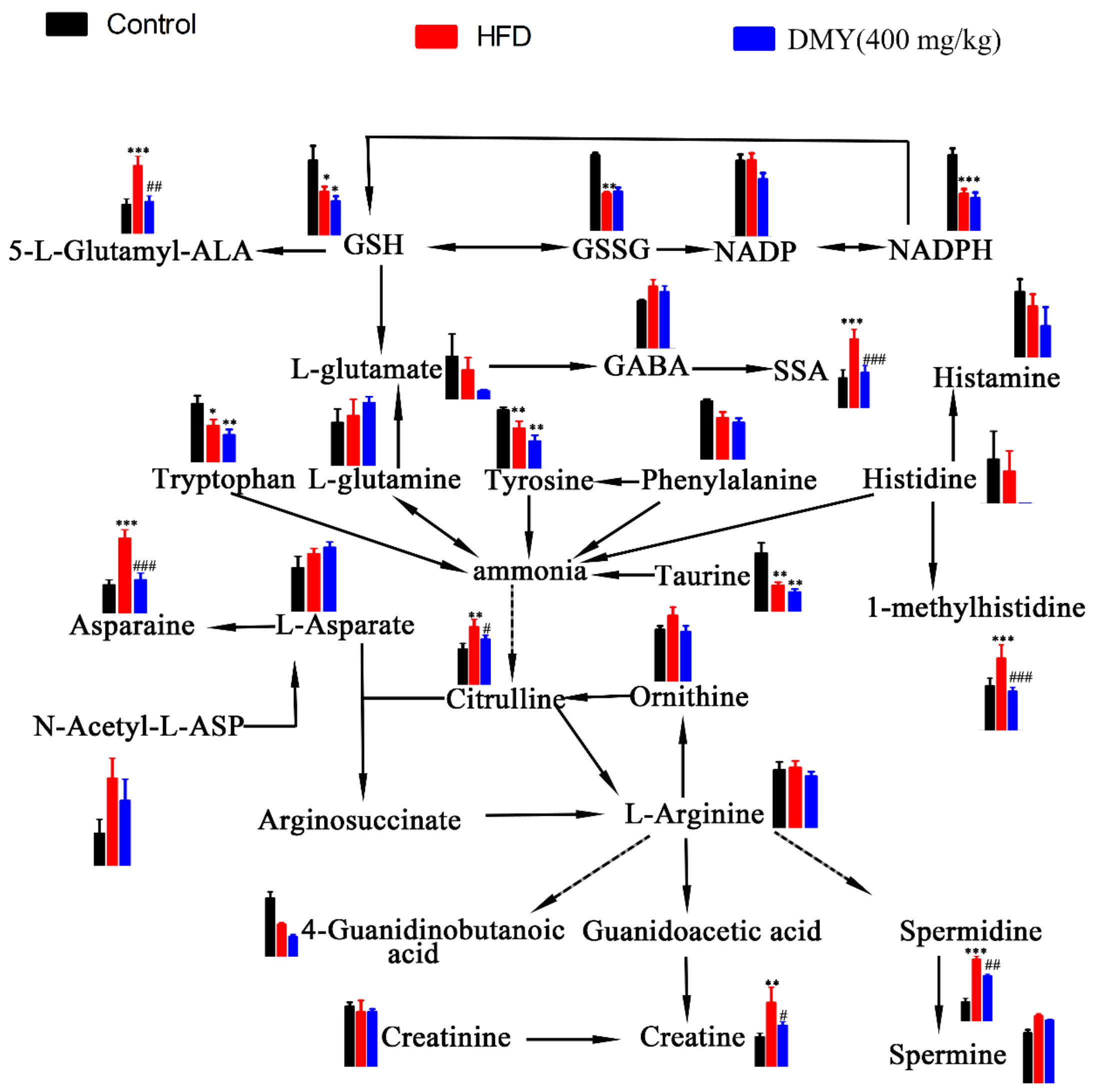

Figure S4. Metabolome pathway map of quantified metabolites, including components of the GSH biosynthesis, amino acids biosynthesis and urea cycle in all groups. * *P<0.05* vs the control group; ** *P<0.01* vs the control group; *** *P<0.001* vs the control group; # *P<0.05* vs HFD model group; ## *P<0.01* vs HFD model group; ### *P<0.001*vs HFD model group, n=6.

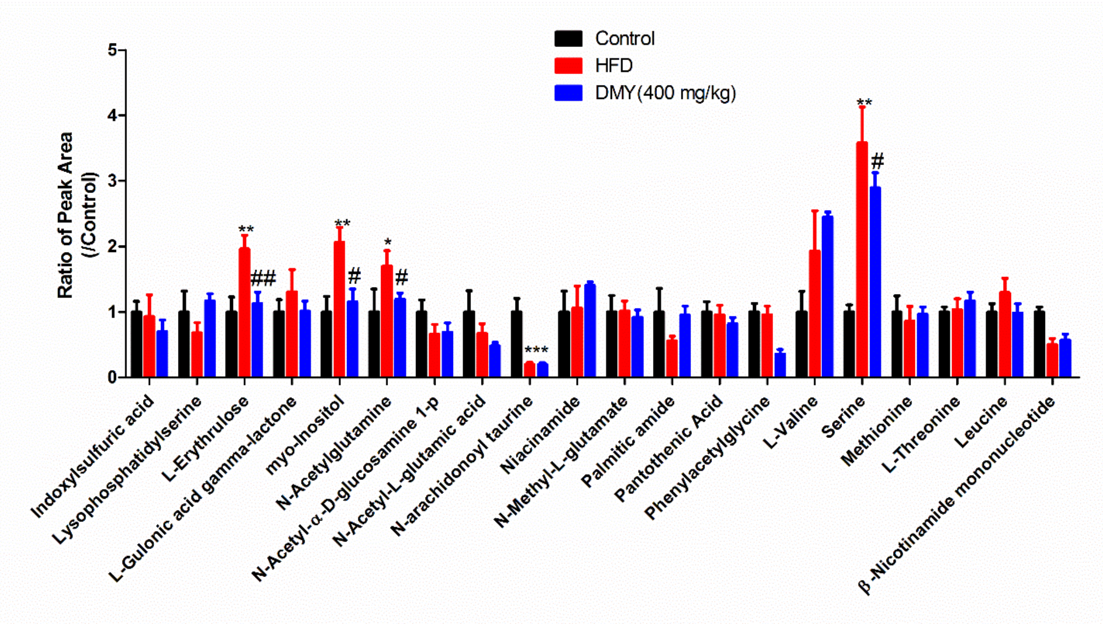

Figure S5. Effect of DMY on metabolic substrates in all groups. * *P<0.05* vs the control group; ***p<0.01* vs the control group; ****p<0.001* vs the control group; # *P<0.05* vs HFD model group; ## *P<0.01* vs HFD model group; ### *P<0.001* vs HFD model group.

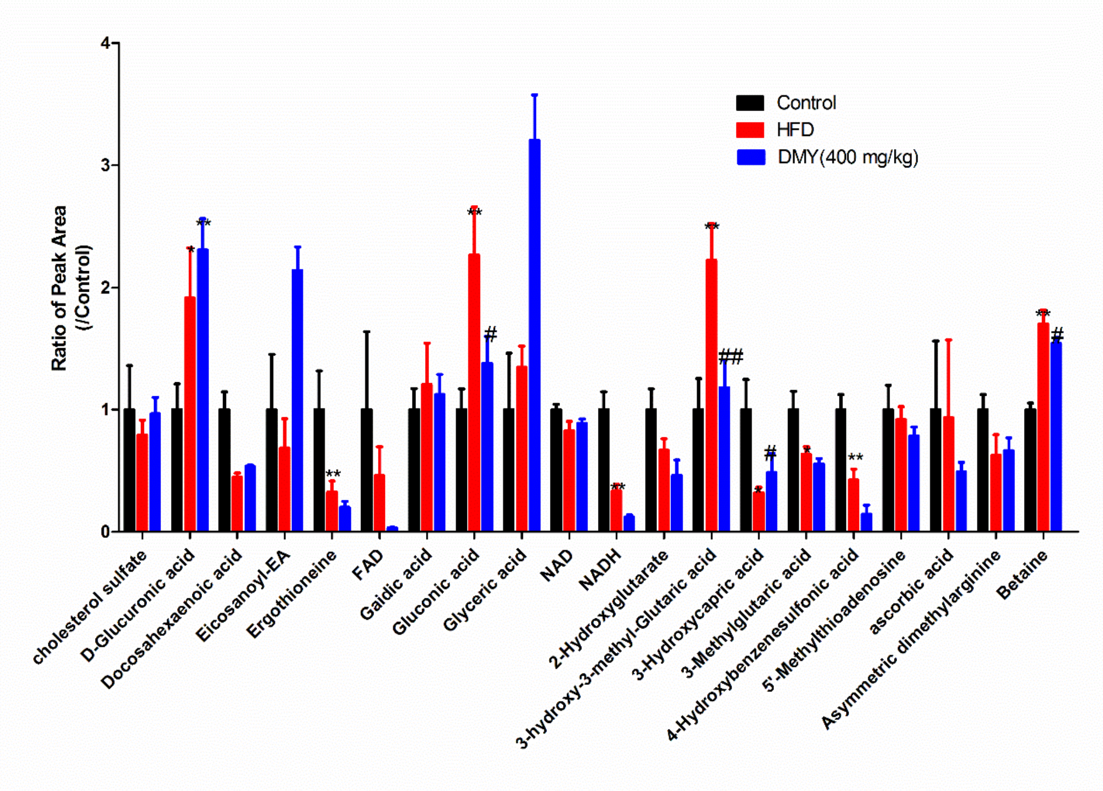

Figure S6. Effect of DMY on metabolic substrates in all groups. * *P<0.05* vs the control group; ***p<0.01* vs the control group; ****p<0.001* vs the control group; # *P<0.05* vs HFD model group; ## *P<0.01* vs HFD model group; ### *P<0.001* vs HFD model group

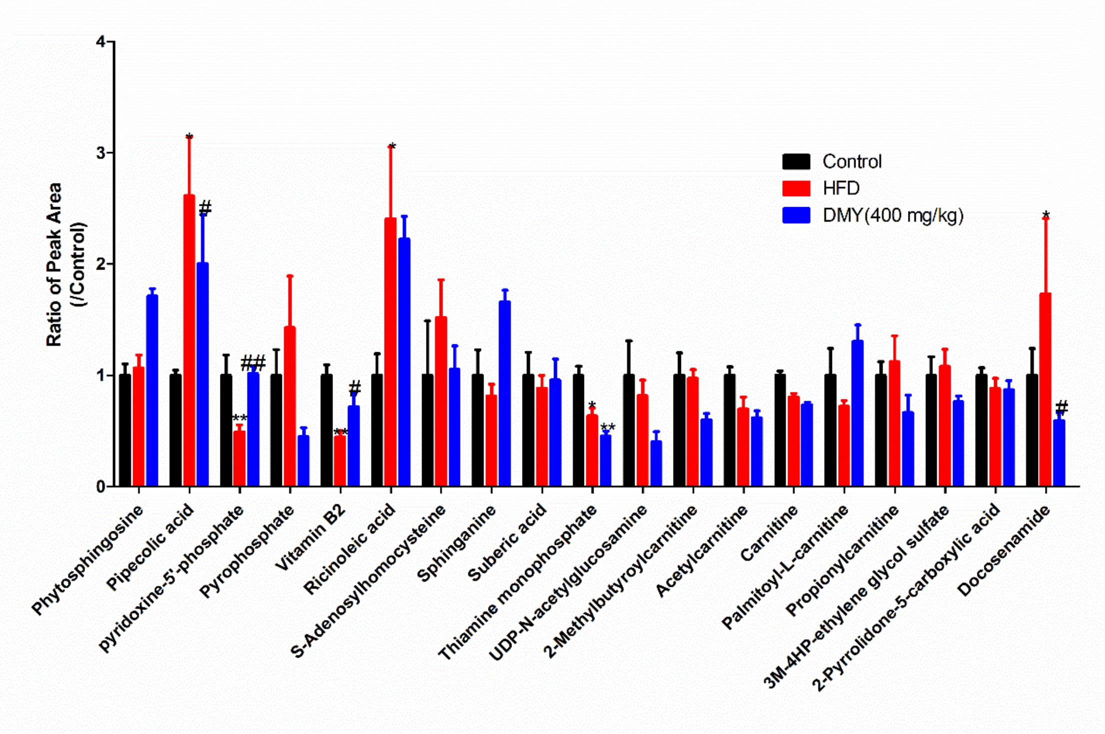

Figure S7. Effect of DMY on metabolic substrates in all groups. * *P<0.05* vs the control group; ***p<0.01* vs the control group; ****p<0.001* vs the control group; # *P<0.05* vs HFD model group; ## *P<0.01* vs HFD model group; ### *P<0.001* vs HFD model grou

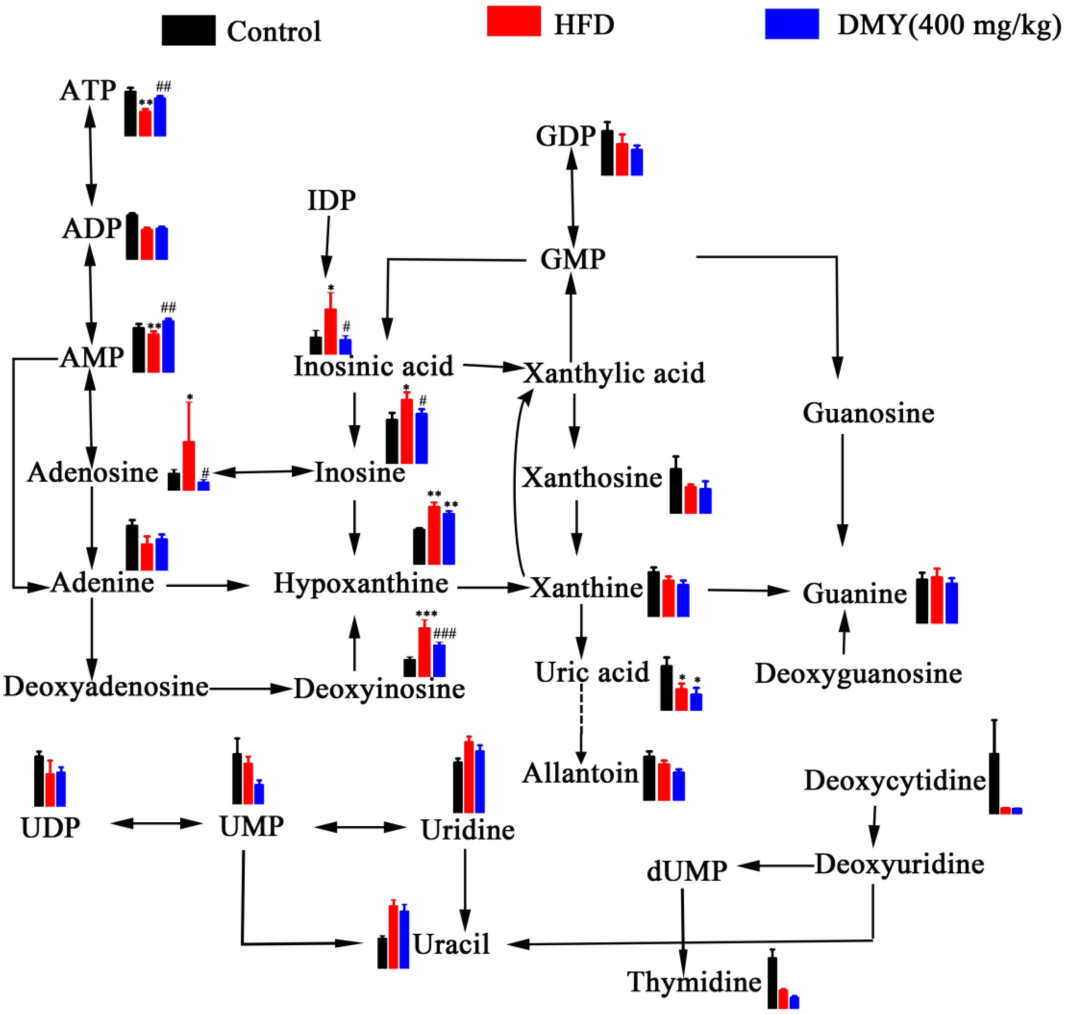

Figure S8. Metabolome pathway map of the quantified metabolites, including the components of purine metabolism and pyrimidine metabolism in each of the groups. * *p<0.05* vs. the control group; ** *p<0.01* vs. the control group; *** *p<0.001* vs. the control group; # *p<0.05* vs. the HFD model group; ## *p<0.01* vs. the HFD model group; ### *p<0.001* vs. the HFD model group, n=6.

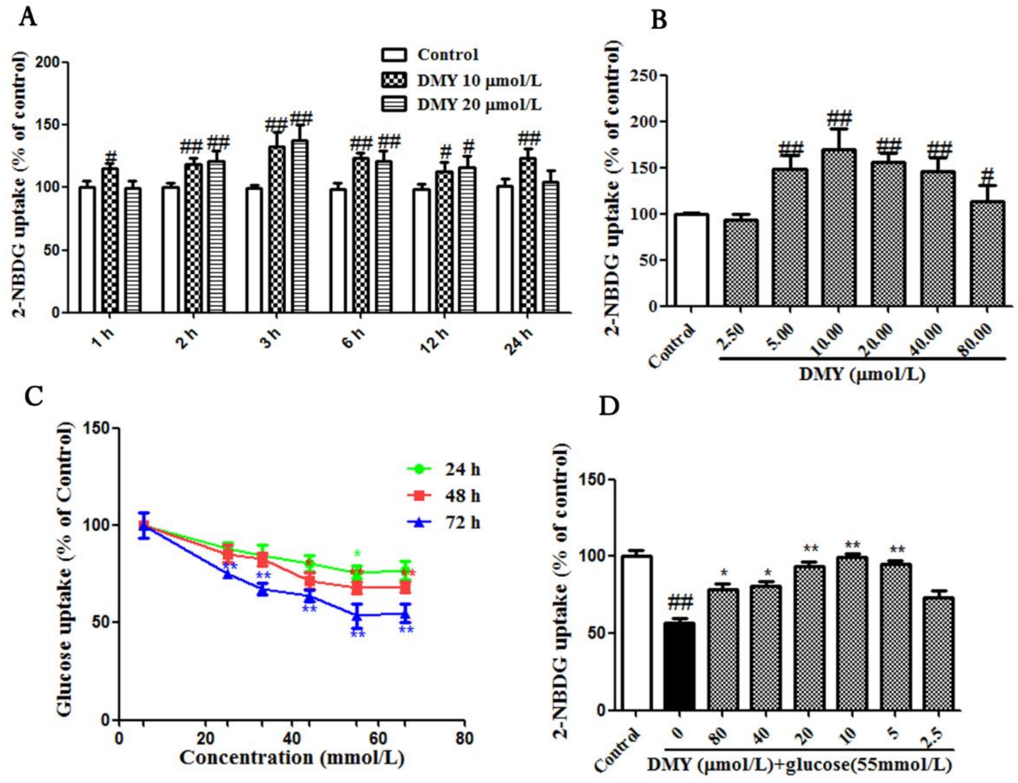

Figure S9. Effects of DMY on 2-NBDG uptake into HepG2 cells. A. Time-dependent effect of DMY on 2-NBDG uptake. HepG2 cells were pretreated with 10 μmol/L or 20 μmol/L DMY for different time. B. Dose-dependent effect of DMY on 2-NBDG uptake. HepG2 cells were incubated with different concentrations DMY for 12 h. C. Dose-dependent and time-dependent effects of glucose on 2-NBDG uptake. D. Pre-protective effect of DMY on insulin resistance induced by high glucose. HepG2 cells were used to detect 2-NBDG uptake after being stimulated for 15 min with 1×10^{-7} mol/L insulin. All experiments were performed at least 3 times. * *P<0.05* vs the control group; ***p<0.01* vs the control group; ****p<0.001* vs the control group; # *P<0.05* vs 55 mmol/L glucose-treated group; ## *P<0.01* vs 55 mmol/L glucose-treated group.

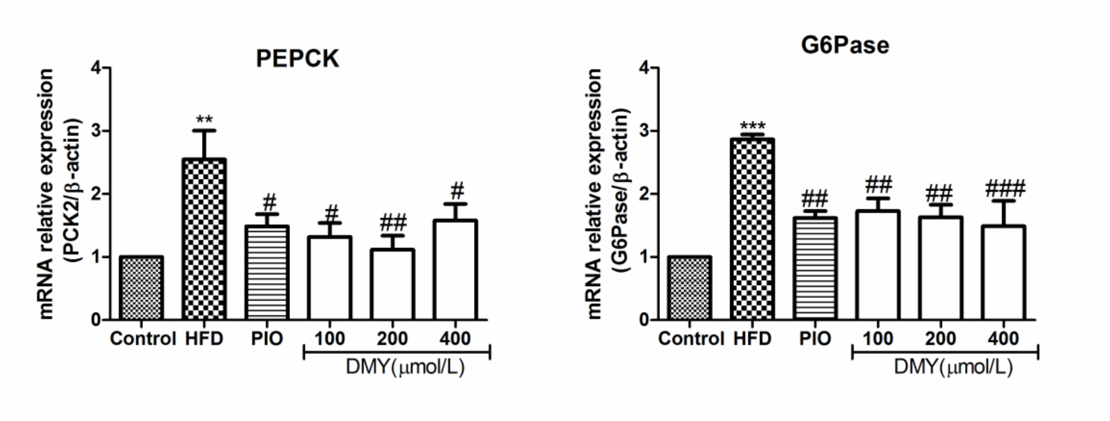

Figure S10 The effects of DMY on mRNA levels of PEPCK and G6Pase genes *in vivo*. (A) Quantitative PCR analysis demonstrated the effect of DMY on the mRNA expression of PEPCK and G6Pase in HFD-fed rats. (n=4). ** *p<0.01* vs the control group; *** *p<0.001* vs the control group; # *p<0.05* vs the

HFD model group; ## *p<0.01* vs the HFD model group; ### *p<0.001* vs the HFD model group.

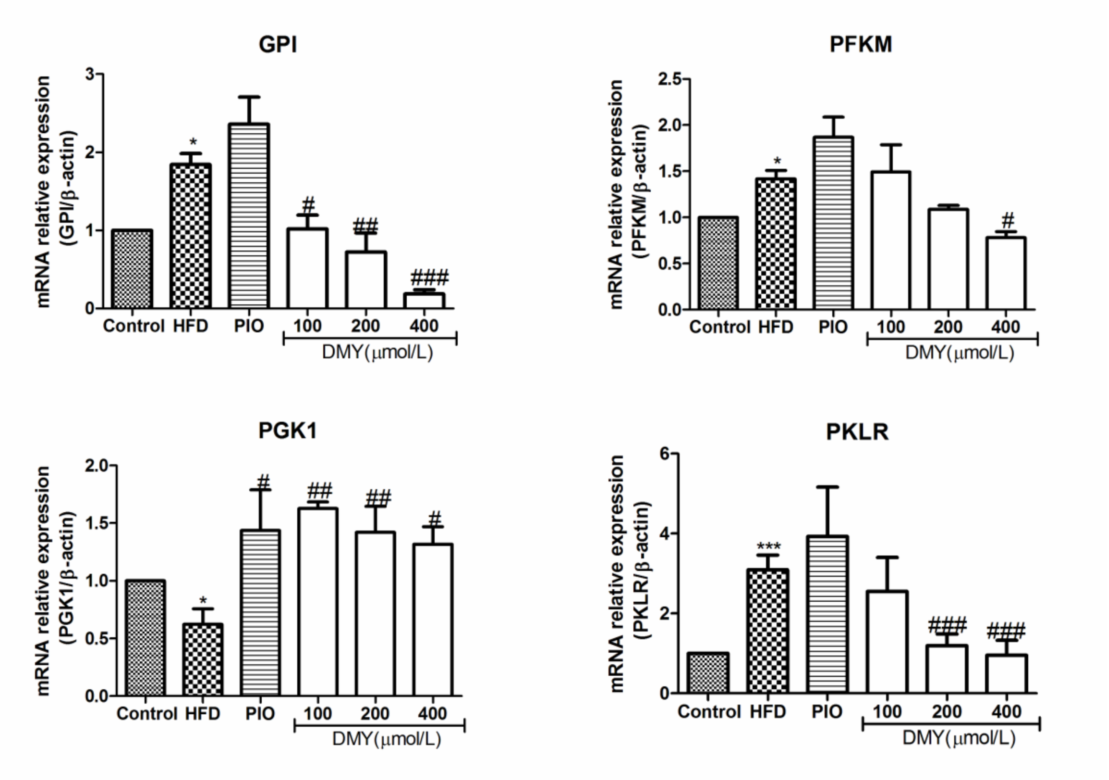

Figure S11 The effects of DMY on mRNA levels of glycolysis-related genes *in vivo*. (A) Quantitative PCR analysis demonstrated the effect of DMY on the mRNA expression of glycolysis-related genes GPI, PFKM, PGK1 and PKLR in HFD-fed rats. (n=4). ** *p<0.01* vs the control group; *** *p<0.001* vs the control group; # *p<0.05* vs the HFD model group; ## *p<0.01* vs the HFD model group; ### *p<0.001* vs the HFD model group.
